# Effects of Land Use on Land Surface Temperature: A Case Study of Wuhan, China

**DOI:** 10.3390/ijerph18199987

**Published:** 2021-09-23

**Authors:** Youpeng Lu, Wenze Yue, Yaping Huang

**Affiliations:** 1 Department of Land Management, Zhejiang University, Hangzhou 310058, China; luyoupeng@zju.edu.cn; 2 School of Architecture and Urban Planning, Huazhong University of Science and Technology, Wuhan 430074, China; hust_hyp@sina.com

**Keywords:** urban heat island, land surface temperature, land use, Wuhan, spatial regression model

## Abstract

In this study, we aim to understand the impact of land use on the urban heat island (UHI) effect across an urban area. Considering the case study of Wuhan, China, land use factors and land surface temperatures (LSTs) of 589 planning management units were quantified in order to identify the spatial autocorrelation of LST, which indicated that a traditional regression would be invalid. By investigating the relationships between land use factors and the LST in summer, based on spatial regression models including the spatial lag model and the spatial error model, four conclusions were derived. First, the spatial error model effectively explains the relationships between LST and land use factors. Second, the impact on LST of the percentage of industrial areas is significant even though the impacts of land cover and building-group morphology indicators are combined, indicating that anthropogenic heat emission of industrial production contributes to high LSTs. Third, the relationship between the percentage of commercial area and LST is significant in the Pearson correlation analysis and traditional regression models, while not significant in spatial error model, suggesting that the urban heat environment of a commercial area is determined by the land use factors of the surrounding area. Fourth, the UHI effect in industrial and commercial areas could be precisely mitigated by not locating industrial areas beside residential areas, and setting up buffer zones between commercial areas and surrounding traditional residential areas. Overall, the results of this study innovatively deepen the understanding of the impact of the percentage of different urban land use types on the urban heat environment at the scale of planning management units, which is conducive to formulating precise regulation measures for mitigating UHI effects and improving public health.

## 1. Introduction

The urban heat island (UHI) effect is a phenomenon in which the temperature in urban areas is higher than that in the surrounding rural areas, and has long been considered to be a significant issue that endangers the health of urban residents [1]. By 2050, it has been predicted that 68% of the world’s population will live in cities [2], and that land use changes brought about by rapid urbanization will affect surface energy balance and airflow, altering urban thermal environments, and thus exacerbating the UHI effect [3,4]. Since urban heat waves and UHI could potentially interact, urban heatwaves caused by the superposition of UHI and climate warming would aggravate air pollution and increase energy consumption, and also easily lead to an increase in human blood thickening and a decrease in visceral blood perfusion [5]. These changes would result in increased cardiovascular burden and seriously threaten the health of residents [6]. Therefore, consciously alleviating these intractable problems in relation to residents’ health and minimizing energy consumption by reducing the UHI effect is key to promoting sustainable development of both nations and cities.

It has been widely acknowledged that land use in urban subsurface is the main cause for an exacerbated UHI effect, as correlations of land cover and three-dimensional (3D) building morphology with the UHI effect have been widely explored. Such correlations are both largely associated with the amount of thermal radiation received at the surface [7,8]. Conventional studies have analyzed the relationships between landscape pattern indexes of land cover and land surface temperature (LST), as well as the relationships between 3D building morphology indicators, for example, building height, building density, floor area ratio, sky visibility, and street height to width ratio, and LST [9,10]. On the one hand, it has been found that large concentrations of impervious surface area exacerbate the UHI effect [11,12,13,14], suggesting a significant correlation between impervious surface area and LST [15,16]. These results illustrate the significant impact of built-up areas on the UHI effect. On the other hand, a compact building layout likewise plays a role in exacerbating the UHI effect, indicated by the fact that building density and height significantly deteriorate the thermal environment of an urban area [17]. Dai, Guldmann, and Hu (2018) identified a significant positive correlation between the normalized difference built-up index (NDBI) and LST [18]. Yang, J. and Jin, S. et al. (2019) suggested that the architectural pattern was one of the important drivers of climate change, which was evidenced by the fact that high-density high-rise buildings increased surface temperatures [13]. In addition, building height and sky visibility significantly affect LST [19]. However, there is disagreement among the studies on the effect of land use indicators on land LST. Some researchers have argued that high-density, low-height building masses have resulted in higher UHI intensities, while others have argued that compact building masses have helped to mitigate the UHI effect [12]. Chun and Guldmann (2014) found that sky visibility was central to LST and had divergent impacts on the UHI intensity in different study areas [9]. In a similar vein, some theses have proven that lower building densities and higher floor area ratios effectively reduced UHI intensity. In contrast, other studies have concluded that lower-density residential buildings generated more heat than higher density residential buildings [20]. This suggests that the effects of land use on UHI intensity and the UHI effect remain controversial and should be subjected to intense scrutiny.

To further investigate the mechanism by which land use affects the UHI effect, existing studies have developed the surface energy balance equation from the perspective of energy conservation. The surface energy balance equation suggests that the surface thermal environment of an urban area is affected by surface heat radiation and that the anthropogenic heat emission is also a significant cause of the UHI effect [21]. Anthropogenic heat is the heat released into the atmosphere due to human activities including transportation; industrial, residential, and commercial buildings; and human activities [22,23]. A simulation of the Yangtze River Delta (YRD) suggested that anthropogenic heat emissions make a considerable contribution to the mean UHI intensity [24,25]. Anthropogenic heat emissions consist of industrial production, building energy consumption, transportation, and human metabolism, which are closely related to human activities [26]. Since human activities are spatially heterogenous, anthropogenic heat emissions differ with respect to urban functional zones, which are closely related to land use type. In an area with certain types of land use, anthropogenic heat is generated during the operation of industrial, commercial, and residential tasks [27]. The spatial and temporal variation of anthropogenic heat emissions mainly result from industrial production, traffic volume, building energy consumption, and population density [28]. The production of a vast amount of anthropogenic heat by industrial production has been reported by studies on the anthropogenic heat emission structure of the YRD, indicating that industry has contributed more heat than other heat sources. In addition, it has been found that LSTs are significantly higher in industrial areas than in other areas [29,30,31]. This phenomenon has been attributed to the large number of anthropogenic heat emissions generated by industrial production [32,33]. In addition, Chapman, S. (2016) suggested that building energy consumption and transportation have important roles in the UHI effect, evidenced by the significant anthropogenic heat emissions in the city center of four Australian capital cities during the daytime, in which the highest emissions were found during working hours [34]. A similar amount of anthropogenic heat emissions in the city center was also observed by another study in Tokyo [35]. A case study of Singapore suggested commercial areas, high-density public housing areas, and low-density residential areas have the highest hourly heat emission intensity [28]. Another similar case study of Toronto further revealed significantly higher average LSTs of commercial and industrial areas, similarly reaching 29.1 °C, higher than government/institutional (27.3 °C) and residential (27.3 °C) areas [36]. It has been concluded that the main sources of anthropogenic heat emissions in urban centers closely correspond to certain types of land use [37]. Thus, the proportion of different land use types may significantly impact the surface thermal environment, in particular, the proportion of industrial and commercial areas.

However, notwithstanding the significant spatial heterogeneity in anthropogenic heat emissions in areas with different types of land use, such an indicator as land use has rarely been highlighted in investigations of the correlation between land use and UHI effect. The neglect of land use type in such correlation studies may be a potential factor contributing to the spatial uncertainty in the effects of land cover and 3D building morphology on UHI intensity. It has been indicated that the core land use indicators affecting UHI intensity differ among sites with different land use types [38]. The effect of 3D building morphology on UHI intensity is more significant in areas with commercial and high-density residential land use types than in other areas. This study demonstrates that land use type indirectly affects land cover and 3D building morphology on the UHI intensity process. However, it is unknown how land use type acts in conjunction with other land use indicators on UHI intensity. Therefore, to comprehensively understand the pathway of land use affecting the UHI effect, it is necessary to investigate the concrete impacts of the proportions of different land use types on the UHI effect.

Methodologically, studies have commonly used regression models to identify the correlation between land use and LST. However, traditional regression models underplay the heat transfer between different samples that originates from the unavoidable air circulation between adjacent units. Such interacting thermal environments of spatial units give rise to spatial autocorrelation of LSTs in different spatial units. As a result, spatial regression models such as the spatial lag model (SLM) and spatial error model (SEM) have been used to deal with these problems [39,40].

With regard to the choice of spatial analytic units, a large number of studies have shown that the choice of different spatial analytic units leads to different correlation results [41]. A spatial grid is commonly employed as the analytic unit for correlations between land use indicators and LST (e.g., 500 × 500 m, 1000 × 1000 m) [42,43]. The main reason for this is that the LST data and some land use data such as land cover are derived from remote sensing raster images, and using a spatial grid consistent with the data source of remote sensing raster images as a spatial analytic unit can retain complete LST information [17,44]. However, a spatial grid mechanically cuts off the original organic building groups, and therefore using a spatial grid to segment a study area does not provide sufficient and complete land use information. At the same time, the conclusions on the correlation between land use and LST derived from using a spatial grid as a spatial analytic unit lack guidance for planning management. In view of this, some researchers have started to adopt the planning management unit as the spatial analytic unit [45,46]. In fact, land use in urban areas is based on the planning management unit as the object of planning and implementation, and the findings of the correlation between land use and LST based on this spatial unit have important practical implications for guiding how to mitigate the UHI effect through land use.

In this study, we aimed to theoretically explore how land use types impact the UHI intensity together with other land use indicators, and to make policy recommendations for urban planning. We chose LST to reflect the relative relations of the UHI effect between the samples, and LST was regarded as the dependent variable. In fact, both atmospheric temperature and LST have their advantages and disadvantages. On the one hand, atmospheric temperature could directly and rapidly reflect anthropogenic heat created by human activities, which is more applicable than LST. Such a point of view is partly supported by the fact that anthropogenic heat plays an important role in daily atmospheric temperature [47]. On the other hand, limited by the number of points for measuring atmospheric temperature, only a small fraction of the urban area could be measured. Since the LST data covered the whole urban area, we could obtain many more samples to exam the relationship between land use and LST, leading to a more objective and accurate result. Therefore, considering that LST has a close correlation to atmospheric temperature and the domain of universal coverability of LST, we chose LST to reflect UHI in this study and achieved relatively accurate results by using the LSTs of 589 planning regulatory units.

As compared with European and American cities, large cities in China face the dual challenges of incremental expansion and urban renewal, which present a more complex urban land use pattern. This study takes Wuhan as a case study, a city with land use characterized by significant spatial heterogeneity and hundreds of water bodies within the city limits [7,48]. Practically, under the influence of the monsoon climate, Wuhan is hot and humid in the summer [17]. In this sense, this case study helps to reveal the comprehensive impact of land use on LST and has obvious practical implications. Further, we adopt the planning management units as the basic analytic unit in this study to propose a planning strategy with practical implications [17,49].

## 2. Materials and Methods

### 2.1. Study Area 

**Figure 1:**
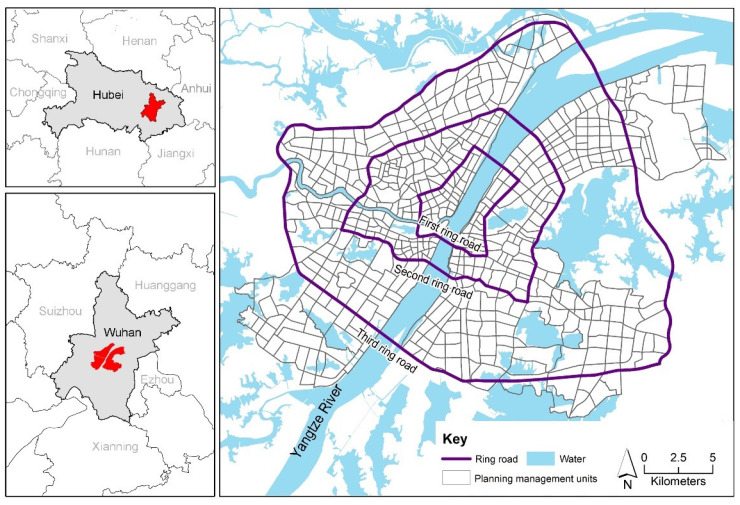
Location of Wuhan city and the study area.

Wuhan is the capital of Hubei province (113°41′–115°05′ E, 29°58′–31°22′ N) and is one of the largest cities in China. Specifically, the core area of Wuhan city was selected as the study area, containing 589 planning management units with an administrative area of 522.68 km^2^ (Figure 1).

### 2.2. Land Surface Temperature (LST) Data Retrieval

The LST data were collected from cloud-free Landsat 8 thermal infrared sensor (TIRS) images on 6 October 2014. The Landsat 8 data were derived from the United States Geological Survey (USGS) at a spatial resolution of 30 m (http://www.usgs.gov/) (accessed on: 6 October 2014). The process of converting the Landsat 8 data to LST was carried out based on the method of a radiative transfer equation using ENVI version 5.1 with three steps. First, a radiometric and geometric correction was processed in the WGS 84 coordinate system and Universal Transverse Mercator projection. Second, the pixel values were converted to brightness temperatures by the RTE method [50]. Third, according to the atmospheric transmission, and the upwelling and dowelling radiance derived from NASA (http://atmcorr.gsfc.nasa.gov) (accessed on: 6 October 2014), a blackbody spectral radiance image and the LST in degrees Celsius for each grid were retrieved in sequence.

### 2.3. Land Use Factors Extraction

According to previous studies [9,51,52,53], this study integrated three domains of land use indicators, including land use type, land cover, and building morphology (Table 1). All the data applied to calculate land use factors were acquired in 2014, consistently presenting the land use situation.

**Figure 2:**
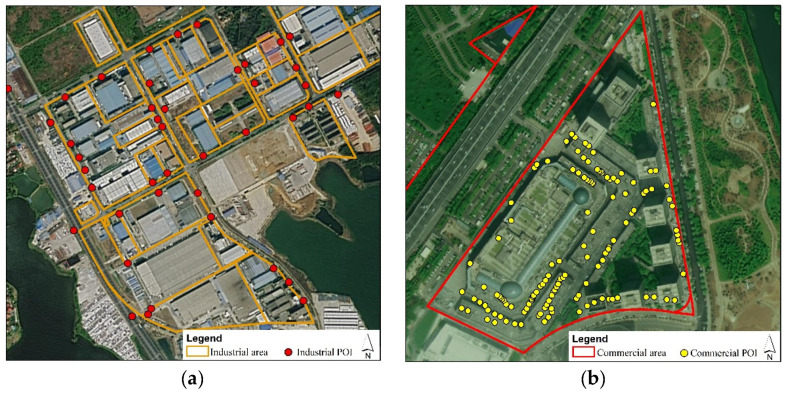
Industrial area (**a**) and commercial area (**b**) boundary derived from the land use map and industrial POI derived from Sina Weibo.

Land use types can be delineated by their spectral and structural features extracted from high-resolution remote sensing images and also by their socioeconomic functions derived from geographic information data [54]. First, traffic networks were applied to divide the blocks. The traffic networks divide a city region into subregions and also block the heat circulation on the surface of a city [55]. Second, the land use types of each block were preliminarily identified according to the land use map (2014) in vector version acquired from the Wuhan Natural Resources and Planning Bureau. The land use map database was set up according to artificial field surveying, considering the overall information of the business registration and cadastral land map, which closely reflected the main activities within each block. Third, we extracted POIs and determined the dominant function of the blocks (Figure 2). The functional intensities of the different POI types were normalized using kernel density estimation. The kernel density analysis was implemented using a quadratic kernel function with a search radius of 500 m [56]. The regions with relatively higher POI point density values indicated the dominant function of the blocks. Four, according to the land use type controversies between the land use map and POI data, we selected the blocks to be corrected. In detail, by comparing the land use types and the dominant functions derived from the land use map and POI data, respectively, we determined the target blocks with land use types that may have been misidentified by the land use map. Five, with the aid of visual interpretation based on high-resolution satellite images (the Google Earth images) and our survey of the study area, we artificially determined the actual and relative land use types of the blocks [57]. The POI data were obtained from the Resource and Environment Science and Data Center (https://www.resdc.cn/data.aspx?DATAID=341) (accessed on: 3 September 2015), and were originally derived from Autonavi. Among the 22 first-level classifications of the POI data, we extracted several types of POI data in relation to industrial and commercial functions. Furthermore, we extracted industrial and commercial areas from the land use type dataset, which probably discharged more anthropogenic heat according to previous studies in the literature. In order to verify the land use type of the blocks with different types of POIs, first, we compared the number of different types of POIs. Then, for the condition that the number of different types of the POIs was even within a block, we further investigated the names of the companies, which could be derived from POI data, to determine the actual and relative primary functions of the blocks. According to the land use map data obtained from the Wuhan Natural Resources and Planning Bureau, 64.33% of the 6325.78 ha of industrial area in the study area belong to the "third manufacturing” (M3) category. The definition of this type of industrial area is an industrial area which could seriously interfere with a surrounding residential area and public environment. Therefore, it is suggested that the industrial area contained light industries and “boiler” industries in the study area.

The land cover data were extracted from Landsat 8 data which were simultaneously consistent with LSTs. The Envi 5.1 software was applied to preprocess the remote sensing images, which followed the steps including atmospheric correction, unified coordinate system, and image cropping of remote sensing raster images. Then, the land use cover classification interpretation used a combination of methods of supervised and unsupervised classification to obtain the spatial distribution of four types of land cover: impervious surface, vegetation, water body, and bare land. Furthermore, the Google Earth platform was employed for manual visually assisted correction combined with high-resolution images. The acquisition time of the high-resolution images was 31 December 2014, with a resolution of 30 m.

The 3D building morphology indexes were calculated based on the building vector dataset. Building density and floor area ratio were employed as the building morphology indexes, which are the proportions of the building base area and building floor area of each planning management unit, respectively.

### 2.4. Regression Analysis

SLM and SEM were conducted if the Moran’s I value of LST showed significant spatial clustering, suggesting that the local LST values were impacted by the surrounding area, and the traditional regression models may not be suitable in the study area. The SLM and SEM equations can be expressed, respectively, as follows [58]:

(1) y=ρWy+Xβ+ϵ

where ρ is a spatial autocorrelation parameter and Wy denotes the spatial weight matrix, X is the matrix of explanatory variables without an intercept term, β is a vector of slopes, and ε is a vector of random error terms.

(2) y=Xβ+γWϵ+δ

where γ is the spatial autocorrelation parameter, Wε denotes the spatial weight matrix, and δ represents a vector of the error terms.

The SLM and SEM models consider the influence of dependent and independent variables in the surrounding area on the explanatory variables of the study area, respectively. Furthermore, a spatial lag term and a spatial error term were applied to the measurement equations of the SLM and SEM models [58]. The tests of the Lagrange multiplier (LM) and the robust Lagrange multiplier (robust LM) indices were used to determine the appropriate regression model [59]. By comparing the LM and robust LM of the SLM and SEM models, the one with stronger spatial dependence was chosen to explain the impact significance of USF factors on LST across the whole study area [60,61].

## 3. Results

### 3.1. Spatial Variation of LST

**Figure 3:**
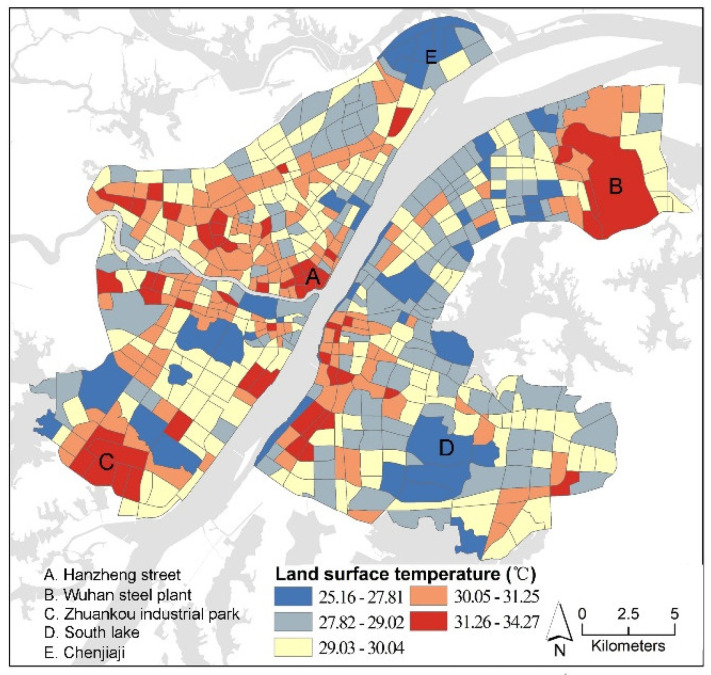
Distribution of LSTs.

The spatial distribution patterns of LSTs are shown in Figure 3. The planning management units were classified according to the average LST. Then, the natural breakpoint method was employed to classify the 589 management units into five categories. The average LST and variance of each category of planning management units are shown in Table 2. It can be observed that the planning management unit with the highest LST is located in the Zhuankou industrial park, which is characterized by concentrated contiguous industrial areas, and reached an LST of 34.27 °C, whereas the management unit with the lowest LST is located around South Lake, with an LST of 25.16 °C. In general, the LST is relatively higher in the core area in relation to the suburb area. In addition to the industrial areas, the high LSTs are more likely located in the central areas, such as Hanzheng Street which contains dense construction areas. Industrial areas in the suburbs also show high LSTs, such as the Wuhan Steel Plant and the Zhuankou Industrial Park, whereas the areas with abundant water and vegetation, such as South Lake and Chenjiaji, have the lowest LST (see Figure 3).

### 3.2. Land Use Factors in Different LST Zones

**Figure 4:**
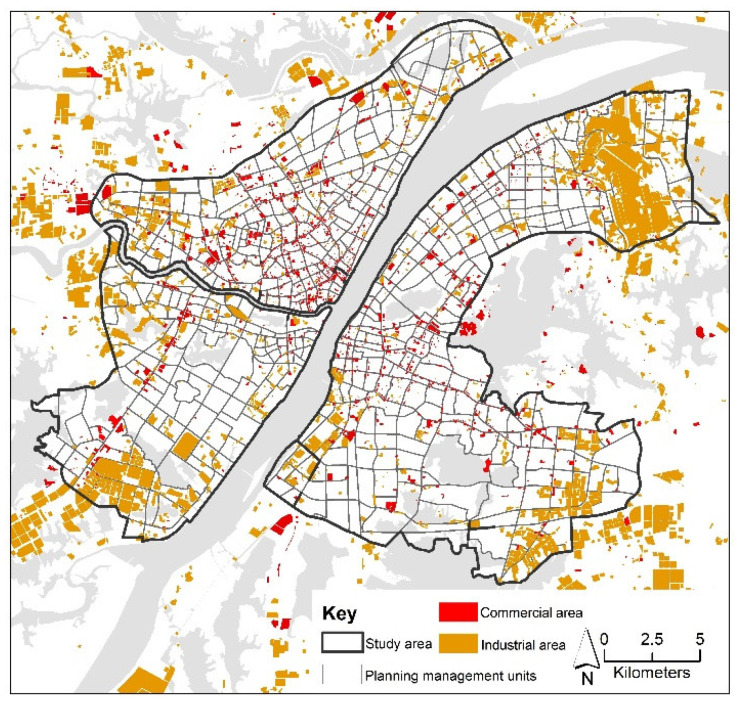
Spatial layout of industrial and commercial areas.

The statistics of each land use indicator in different LST zones are presented in Table 2. Zones 1 to 5 represent the five planning management categories, with different LST section rankings from high to low. Specifically, PIA (percentage of industrial area) and PCA (percentage of commercial area) were calculated according to the spatial distribution of industrial and commercial areas shown in Figure 4. The statistics indicate that the mean values of PIA, ISA (percentage of impervious area), PW (percentage of water), PV (percentage of vegetable), and BD (building density) in different LST zones showed regular changes with the variation of LST. PIA, PCA, ISA, and BD increase with increasing LST, and the proportions of these land use indicators were significantly higher in the higher temperature zones than in the lower temperature zones. For instance, PIA is as high as 26.74% in Zone 1, which has the highest LST, while it is only 2.36% in Zone 5. In contrast, PW and PV decrease with increasing LST, indicating a higher percentage of vegetation and water bodies in the lower temperature zones. PW is as high as 28.76% in Zone 5, where the temperature is lowest, while it was only 3.44% in Zone 1. However, the variation in FAR (floor area ratio) across LST zones is erratic, and it does not show a strictly unidirectional increase or decrease in value with increasing LST, which indicates that the FAR of Zone 1 is lower than that of Zone 2.

PIA, PCA, PW, and PV represent the percentage of industrial area, commercial area, water area, and vegetation area, respectively; ISA represents the percentage of impervious surface area; BD represents building density; FAR represents the floor area ratio.

### 3.3. Spatial Correlation

Global Moran’s I was applied to verify the LST spatial autocorrelation hypothesis. The Moran’s I value for LST is 0.3043 (*p*-value < 0.001). This result suggests a significant spatial clustering for LST in different seasons, meaning OLS models are not appropriate. Accordingly, a spatial regression analysis, including SLM and SEM, was required in our study area. Furthermore, an LM test and robust LM test were applied to select the relatively appropriate model among the SLM and SEM models. The results are displayed in Table 3, suggesting that the SEM models were larger than that of the SLM models, indicating that SEM performed better than SLM. Hence, we purposefully employed SEM to further explore the correlation of USF factors and LST.

### 3.4. Correlation between Land Use Factors and LST

To initially verify the correlation between each land use indicator and LST, we used SPSS software to perform a Pearson correlation analysis on each indicator (Table 4). We found that all seven selected land use indicators showed significant correlations with LST. Among the indicators of land use types, PIA and PCA were significantly positively correlated with LST, which indicated that industrial and commercial functions are each significantly correlated with the UHI effect. Among the indicators of land cover, except for ISA, which had a significant contribution to LST, all other land cover types were significantly negatively correlated with LST. The building morphology indicators both showed significant positive correlations with LST, indicating that the growth of both two-dimensional and 3D buildings has a significant contribution to the UHI effect. Among the land use indicators significantly correlated with LST, BD has the highest correlation coefficient with LST, up to 0.598, followed by PIA, ISA, PW, and PV, with the coefficients ranging from 0.426 to 0.472. Although PCA, FAR, and LST were also significantly associated, the correlation coefficients were lower. Subsequently, we used multiple linear regression to identify the co-collinearity between several land use indicators and LST, and the results showed that the VIF values of each indicator were below 10, indicating that there was no co-collinearity between the above indicators.

After removing the autocorrelation effect, we used the SEM model to determine the relationship between the land use factors and LST, as shown in Table 5. The model showed that all land use indicators were significantly correlated with LST except PCA and ISA. PIA and BD were significantly and positively correlated with LST, while PV, PW, and FAR were significantly negatively correlated with LST. This result is slightly different from the results of the Pearson correlation analysis, mainly in that PCA and ISA were no longer significantly correlated with LST in the SEM model, which indicates that the influences of PCA and ISA on the LSTs of management units were weakened after the SEM model considered the influence of explanatory variables of adjacent spaces on the LST of each management unit. Among the various land use indicators, the influences of building morphology indicators on LST were greater, as shown by the top two absolute values of the coefficient of influence of BD and FAR on LST, followed closely by PW and PIA as compared with the least influential, which was PV.

In Table 5, we compare the results of OLS and SEM to verify the advantage of the SEM model over OLS model. We found that the SEM model could better fit to LST than the OLS model, as the R^2^ of the SEM model was 0.766, while that of the OLS was only 0.624. The two models tested whether the correlations between the same land use indicators and LST were significant, and the measured results showed that the influencing direction and significance of the impacts of PW, PV, and BD on LST were consistent. BD has a higher coefficient of influence on LST than other indicators in both models, followed by FAR. The coefficients of two land use type indicators, PIA and PCA, have a smaller influence on LST in both models. However, PCA and ISA are significantly associated in one model but not in another. PCA is significantly positively associated with LST in OLS, but it is no longer significant in the SEM model. This result suggests that the OLS model overestimates the effect of PCA on LST.

Subsequently, we used different combinations of land use indicators as independent variables to further test the effects of PIA and PCA on LST, and the results are shown in Table 6. All six models were designed to analyze the effects of industrial and commercial land use on LST. Models 1 and 4 show that both industrial and commercial area uses were significantly positively related to LST. The impact of industrial areas on LST is greater, suggested by the fact that the impact coefficient of PIA in Model 1 is higher than the impact coefficient of PCA in Model 4. Subsequently, we add the land cover indicators and building morphology indicators into the model to identify the impact of PIA and PCA on LST. Models 2 and 3 show that after introducing other types of land use indicators, the coefficient of the impact of PIA on LST decreases slightly, while its contribution to LST is still significant. In particular, the coefficient of the PIA on LST decreases from 0.104 in Model 1 to 0.053 in Model 2 after combining the building morphology indicators, while it only decreases to 0.084 after combining the land cover indicators. This indicates that the anthropogenic heat emissions from its functional operation have a significant contribution. Models 5 and 6 analyze the combined effect of PCA with land cover category indicators and building morphology category indicators on LST, respectively. We found that although PCA is significantly associated with LST in Model 4, the effect of commercial land use on LST is no longer significant when either land cover or building morphology indicators are introduced into the model. Further, the R^2^ values of Models 5 and 6 are 0.672 and 0.595, respectively, both of which are much higher than the 0.383 of Model 4. These results indicate that the contribution of PCA to LST is limited, and the association between commercial area use and LST is mainly due to the companied high proportion of impervious surface and building density, rather than the difference between the commercial function and other land use functions.

## 4. Discussion

### 4.1. Impact of Land Use Type Factors on LST

Previous studies have attempted to establish a link between the theory of interaction of various land use indicators and thermal environment and the policy of landscape and urban planning. It has been widely demonstrated that increasing surface greenery, reducing building density, and optimizing 3D building morphology indicators such as sky view factor, can play a role in reducing the UHI effect. Therefore, the optimization of land use indicators related to land cover and building morphology is considered to be beneficial in regulating urban microclimate. In this study, we suggest the percentage of specific land use types also plays an important role in the correlation between land use and LST, while many existing studies have ignored such influence.

The significant positive correlation between PIA, PCA, and the mean LST of the planning management units was supported by the Pearson correlation analysis and, additionally, by the SEM model combining land cover and 3D building morphology indicators. Further, the study applied several SEM models to examine the combined effects of other land use indicators with PIA and PCA on LST, and therefore to verify the independent impacts on LST. The results of these models suggest that a consistent and stable positive correlation only existed between PIA and LST when different combinations of indicators were used in the regression models. Such results may be due to the fact that the industrial production process makes extensive use of combustion facilities such as melting furnaces and boilers. During this period, the unutilized fuel is converted into a high-temperature flue airflow, forming waste heat that is discharged to the external environment through chimneys, radiators, and cooling water. Therefore, high PIA levels usually accompany extensive chimney heat discharge, radiator heat discharge, and cooling water heat discharge. Additionally, metal panels are more widely used on the surface of industrial buildings, which could quickly absorb short-wave radiation through radiative heat exchange. In contrast, buildings in other types of functional sites are dominated by masonry materials, which have lower rates of radiative heat exchange. Thus, due to the continuous heat production of industrial production and the rapid heat absorption characteristics of building skins, industrial sites significantly contribute to the UHI effect.

The significance of PCA and LST only appears in the Pearson correlation analysis, while the correlation between PCA and LST is no longer significant in the SEM model when it is loaded with land cover indicators or building morphology indicators. Interestingly, PCA significantly correlates with LST in the OLS model. The significant correlation in the Pearson test results demonstrates that a large proportion of a commercial area is usually associated with relatively high LSTs. Furthermore, the OLS model demonstrates that commercial functions still contribute significantly to LST after considering the effects of various land use indicators such as land cover and building morphology on LST. However, this significant correlation disappears in the SEM model. Such a result suggests that the contribution of commercial function to the UHI effect is overestimated in the OLS model. When the role of surrounding explanatory variables on LST in a particular area are taken into consideration by the SEM model, we found that the contribution of commercial function to the UHI effect was actually insignificant as compared with other land use indicators. This result is understandable as areas with a high percentage of commercial land use are usually surrounded by traditional residential areas characterized by a high percentage of impervious surfaces and high building densities. The SEM model and the OLS model results reveal that the land use indicators of the areas surrounding the commercial areas explain the LST of the commercial areas. This indicates that the land use of the traditional residential areas adjacent to the commercial areas affects the UHI intensity of the commercial areas. This phenomenon could be more obvious when large and dense residential areas surround relatively small commercial areas, where this heating effect on the commercial areas is severely superimposed, resulting in severe heat in the commercial areas. Therefore, the significant positive impact of PCA on LST shown by the Pearson correlation analysis and the OLS model, does not prove a causal relationship between the two. The reason for this positive association revealed by the SEM model is that land use indicators in the surrounding area are important explanatory variables for LST in commercial areas.

### 4.2. Comparison of the Relationships of Land Cover and Building-Group Morphology on LST

The land cover and building-group morphology reflect the 2D and 3D characteristics of the land use, respectively. The OLS and SEM models both indicated that, as compared with land cover, the correlations between LST and building-group morphology indicators were more significant (Table 5). Such results were verified by the fact that the correlation coefficients of the building-group morphology indicators were larger than that of land cover in the OLS and SEM models. The significant contributions of the building-group morphology indicators are displayed in Table 6 and suggest that the participation of the building-group morphology indicators improved more goodness of the models. Such an advantage of the 3D land use indicators over 2D indicators is consistent with several previous studies [9,62,63]. Three-dimensional land use indicators represent a mass of vertical building elevations, which may be directly perpendicular to the direction of solar radiation and efficiently absorb solar radiation. In addition, 3D land use indicators also indicate the amount of artificial material with large heat storage capacity. Therefore, the energy absorbed by the vertical building elevations could be stored and promote the UHI.

### 4.3. Relationships between the Industrial and Commercial Anthropogenic Heat and LST

The pathway of the land use type affecting the urban thermal environment mainly depends on the anthropogenic heat emissions created by industrial and commercial activities. Since the anthropogenic heat emissions in urban centers closely correspond to certain land use types, we selected the industrial areas and commercial areas to investigate the correlations between land use types and urban thermal environment. Previous studies have supported that industrial and commercial areas were the main sources of anthropogenic heat emissions, and anthropogenic heat played an important role in daily atmospheric temperature [47]. Therefore, the influence mechanism of anthropogenic heat emissions from industrial and commercial areas on LST should be further investigated.

Some of the anthropogenic heat produced by industrial and commercial activities is consumed inside buildings; therefore, only some of the total anthropogenic heat is discharged into the air. Nevertheless, since building energy use accounts for a significant fraction of the anthropogenic heat discharge, anthropogenic heat discharge is consistent with building energy use in terms of the spatial pattern [64], which leads to a significant spatial distribution of the air temperature. Meanwhile, due to the continuous energy transfer between surface air and land surface, the air temperature and LST significantly correlated with each other; the consistency was evidenced by a good linear correlation between them [65]. Therefore, we suggest anthropogenic heat emissions and LST are indirectly correlated, which leads to the significant quantitative correlation between the percentage of industrial and commercial areas and LST in our studies. Such a correlation was proven by the fact that energy consumption is positively correlated with LST intensity in the case study of the Yangtze River Delta Urban Agglomeration [66,67].

### 4.4. Planning Strategies Implication

**Figure 5:**
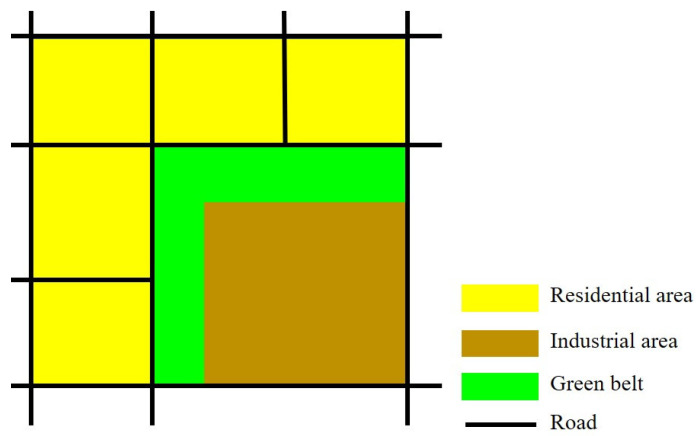
Planning strategies for the spatial layout of industrial areas.

On the basis of the above correlation analysis of PIA, PCA, and LST, we propose some planning strategies to optimize the spatial layout of industrial and commercial functions that are aimed at mitigating the UHI effect. Since there is a significant positive correlation between PIA and LST, the layout of industrial areas adjacent to residential areas and various public facilities should be avoided in order to prevent the adverse effect on residential areas of high-temperature airflow formed by industrial production. Green belts should be set up between industrial areas and other functional spaces for existing cases where industrial areas are adjacent to residential areas and public facilities (Figure 5). The green belts could help to efficiently block the disorderly diffusion of high-temperature airflow generated in industrial areas and to prevent the surrounding urban functions from being heated.

**Figure 6:**
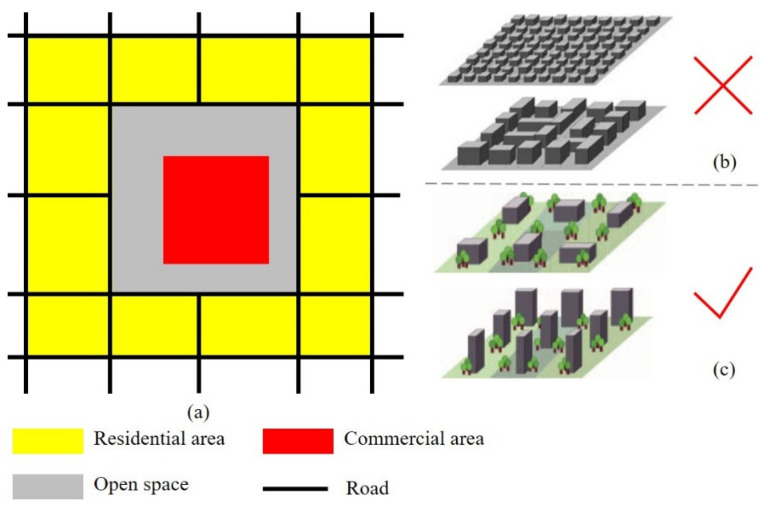
Planning strategies for the spatial layout of a commercial area: (**a**) The ideal special relationships between a commercial area and a traditional residential area, an open space is located between them; (**b**) traditional residential area, which is similar to LCZ 2 (compact mid-rise) and LCZ 3 (compact low-rise); (**c**) optimized residential area, which is similar to LCZ 4 (open high-rise) and LCZ 4 (open mid-rise).

Since the high temperature of commercial areas is mainly caused by the land use of the surrounding areas, attention should be paid to controlling the land use surrounding commercial areas. The land use index of the surrounding areas should achieve the purpose of cooling the commercial areas. On the one hand, extra attention should be paid to the layout of open space around commercial areas. Due to the deteriorative thermal environment of the traditional residential area, which is similar to local climate zone (LCZ) 2 (compact mid-rise) and LCZ 3 (compact low-rise) [51] (Figure 6a), we suggest a buffer zone between a commercial area and surrounding traditional residential areas that aims to prevent the commercial areas from being heated. On the other hand, the land use indicators surrounding the commercial areas should be controlled to avoid increasing the UHI of commercial areas, such as the building density, the percentage of impervious surface, the volume ratio, and so on. The redevelopment of the traditional residential area should optimize the land use indicators by the standards of LCZ 4 and LCZ 5, which are characterized by open high-rise buildings and open mid-rise buildings (Figure 6b).

## 5. Conclusions

As a result of human activities modifying the underlying surface, land use has a profound impact on the spatial variation of the UHI effect. The diversity of human activities in urban built-up areas has created a complex land use situation, and the coexistence of multiple land use types within a given spatial unit is one of the important manifestations of this complexity. However, existing studies in the literature on land use and the UHI effect have focused on the effects of land cover and 3D building morphology on UHI intensity, ignoring the role of land use types on the thermal environment of built-up areas. In fact, the urban thermal environment is a very complex system with multiple influencing factors. A comprehensive recognition and measurement of land use, as well as a multi-perspective analysis of the impact of land use on the thermal environment, would help to gain a deeper understanding of the driving mechanisms of the UHI effect in urban built-up areas and the causes of its spatial variation. In this study, we take Wuhan, China as an example and use spatial regression to analyze the impact of land use factors on the thermal environment. Specifically, we examine the influence of the proportion of industrial area and commercial area on LST to determine the warming effect created by anthropogenic heat. The results of this study indicate that multiple land use indicators, including the proportion of industrial and commercial areas, could explain the spatial variation of LST by using the SEM model. By comparing the correlations between the land use indicators and LST under different combinations, we found that the proportion of the industrial area has a persistent and significant positive correlation with LST, demonstrating the contribution of the industrial production function to the UHI effect. The significant positive correlation of the proportion of commercial area to LST only appears in the OLS model but disappears in the SEM model. This finding proves that the high-temperature phenomenon in commercial areas could be explained by the land use indicators of the surrounding area. In addition, unlike other studies that have used a spatial grid as a sample for correlation analysis, the analysis of the correlation in this study is based on the planning management units. Since such a spatial unit is the basic object of urban planning regulation strategies, the correlation conclusions drawn using this spatial unit are more relevant for land use planning measures to mitigate the UHI effect.

Although this study comprehensively reveals the combined effects of various types of land use indicators on the UHI effect, limitations still exist. First, the production activities in industrial areas vary significantly in the degree of anthropogenic heat emissions due to different production processes and energy consumption. The concrete correlation rules between LST and different types of industrial areas, which are different in the production activities, still remain unknown. Second, as the heat created by commercial activities mainly occurs indoors and is consumed inside the buildings, the urban heat island effect of the commercial areas may be underestimated. Third, many other heat sources in addition to industrial and commercial areas, such as traffic, are also determined by land use systems, while, in this study, we did not discuss the pathway of other land use types and human activities influencing the UHI effect. In addition, correlation measures based on the SEM model suggest that the thermal environment of commercial areas needs to be explained by the land use indicators of the surrounding area. However, it is not clear which characteristics of land use around commercial areas lead to an increase in the temperature of commercial areas. Finally, whether the conclusions regarding the correlations between land use and LST determined based on Wuhan city as a case study can be applied in other cities as well requires further validation.

Some further research is needed to be continued in this field. Considering the different planning strategies for the industrial and commercial areas, further studies should make efforts to identify urban functional units according to land use types. In different urban functional units, we should carry out different planning strategies in line with the correlation between land use types and LST. In order to unfold the complex relationships between land use and the UHI effect, more attention should be paid to closely determine the land use classification. Regarding relying on a manual check for correcting the land use map, we suggest the employment of the POI data should be further investigated. In addition, although atmospheric temperature and LST are closely correlated, we suggest subsequent studies should explore the relationships between land use type and the urban thermal environment applying air temperature.

## Figures and Tables

**Table 1:** The abbreviation and description of the land use indicators.

Categories	Factors	Abbreviation	Description	Data Resource
Land use type composition	Percentage of industrial area	PIA	Measures the proportional abundance of industrial area in the planning management units	Wuhan Natural Resources and Planning Bureau (2014) Resource and Environment Science and Data Center (https://www.resdc.cn/data.aspx?DATAID=341) Autonavi (accessed on: 3 September 2015) *
	Percentage of commercial area	PCA	Measures the proportional abundance of commercial area in the planning management units	Wuhan Natural Resources and Planning Bureau (2014) Resource and Environment Science and Data Center (https://www.resdc.cn/data.aspx?DATAID=341) Autonavi (accessed on: 3 September 2015) *
Land cover	Percentage of impervious surface area	ISA	Measures the proportional abundance impervious surface area in the planning management units	United States Geological Survey (USGS) (earthexplorer.usgs.gov/) (accessed on: 6 October 2014)
	Percentage of water	PW	Measures the proportional water bodies in the planning management units	United States Geological Survey (USGS) (earthexplorer.usgs.gov/) (accessed on: 6 October 2014)
	Percentage of vegetation	PV	Measures the proportional vegetation in the planning units	United States Geological Survey (USGS) (earthexplorer.usgs.gov/) (accessed on: 6 October 2014)
Building-group morphology	Building density	BD	Divide the total base area of all the buildings in the planning management units	Wuhan Natural Resources and Planning Bureau
	Floor area ratio	FAR	Divide the total floor area of all the buildings in the planning management units	Wuhan Natural Resources and Planning Bureau

* The POI data applied in this study were obtained from Resource and Environment Science and Data Center (https://www.resdc.cn/data.aspx?DATAID=341) (accessed on: 3 September 2015) and were originally derived from Autonavi.

**Table 2:** Land use factors in different LST zones.

	PIA	PCA	ISA	PW	PV	BD	FAR
Zone 1 (>31.26 °C)	26.74%	7.95%	92.01%	0.82%	3.44%	0.34	1.07
Zone 2 (30.05–31.25 °C)	10.14%	7.28%	85.44%	1.14%	6.20%	0.25	1.1
Zone 3 (29.03–30.04 °C)	7.24%	4.66%	77.37%	2.61%	11.75%	0.19	0.99
Zone 4 (27.82–29.02 °C)	3.76%	3.85%	71.75%	5.90%	16.35%	0.16	0.89
Zone 5 (<27.81 °C)	2.36%	2.84%	48.73%	22.18%	28.76%	0.09	0.48

**Table 3:** Lagrange multiplier (LM) diagnostics for spatial dependence.

	D.F.	Value	*p*-Value
LM (SLM)	1	153.4246	0.0000
Robust LM (SLM)	1	16.5381	0.0001
LM (SEM)	1	195.5782	0.0000
Robust LM (SEM)	1	58.6917	0.0000

LM represents Lagrange multiplier. SLM and SEM represent spatial lag model and spatial error model respectively. D.F. is the degree of freedom.

**Table 4:** Pearson correlation coefficients of the land use factors and LST.

	Land Use Factors	Pearson Correlation Coefficients
Land use types	PIA (%)	0.426 **
	PCA (%)	0.215 **
Land cover	ISA (%)	0.472 **
	PW (%)	−0.453 **
	PV (%)	−0.436 **
Building morphology	BD	0.598 **
	FAR	0.216 **

** Represents a significance level at 0.001. PIA, PCA, PW, and PV represent the percentage of industrial area, commercial area, water area, and vegetation area, respectively; ISA represents the percentage of impervious surface area; BD represents building density; FAR represents the floor area ratio.

**Table 5:** Comparison of OLS and SEM.

	SEM	OLS
PIA	0.0438864 **	0.230 **
PCA	0.00372912	0.123 **
ISA	0.0109246	−0.259 **
PW	−0.0687816 **	−0.281 **
PV	−0.0309925 **	−0.355 *
BD	0.136333 **	0.734 **
FAR	−0.0838769 **	−0.439 **
R^2^	0.765939	0.624
LL	1477.276743	1376.74
AIC	−2938.55	−2737.48
SC	−2903.53	−2702.45

** Represents a significance level at 0.001. * Represents a significance level at 0.01. SEM represents spatial error model. OLS represents ordinary least squares. PIA, PCA, PW, and PV represent the percentage of industrial area, commercial area, water area, and vegetation area, respectively; ISA represents the percentage of impervious surface area; BD represents building density; FAR represents the floor area ratio. The LL represents log likelihood; AIC represents the Akaike information criterion; and SC represents the Schwarz criterion.

**Table 6:** SEM (spatial error model) results.

	Model 1	Model 2	Model 3	Model 4	Model 5	Model 6
PIA	0.104152 **	0.0532416 **	0.0835761 **			
PCA				0.0215723 *	0.000783343	0.595001
ISA			0.0433368 **			0.0479207 **
PW			−0.0738547 **			−0.0800255 **
PV			−0.0147743			−0.0192404
BD		0.167664 **			0.194329 **	
FAR		−0.0591297 **			−0.078539 **	
R^2^	0.506609	0.695894	0.666540	0.388342	0.671549	0.595001
LL	1256.369315	1404.785989	1371.161039	1195.782597	1380.489894	1312.004322
AIC	−2508.74	−2801.57	−2732.32	−2387.57	−2752.98	−2614.01
SC	−2499.98	−2784.06	−2710.43	−2378.81	−2735.47	−2592.12

** Represents a significance level at 0.001.* represents a significance level at 0.05. PIA, PCA, PW, and PV represent the percentage of industrial area, commercial area, water area, and vegetation area, respectively; ISA represents the per-centage of impervious surface area; BD represents building density; FAR represents the floor area ratio. The LL repre-sents log likelihood; AIC represents the Akaike information criterion; and SC represents the Schwarz criterion.

## Data Availability

The POI data applied in this study were obtained from Resource and Environment Science and Data Center (https://www.resdc.cn/data.aspx?DATAID=341) (accessed on: 3 September 2015), and were originally derived from Autonavi. Land cover data were obtained from United States Geological Survey (USGS) (earthexplorer.usgs.gov/) (accessed on: 6 October 2014). Land use maps were obtained from Wuhan Natural Resources and Planning Bureau.
